# AI in 2D Mammography: Improving Breast Cancer Screening Accuracy

**DOI:** 10.3390/medicina61050809

**Published:** 2025-04-26

**Authors:** Sebastian Ciurescu, Simona Cerbu, Ciprian Nicușor Dima, Florina Borozan, Raluca Pârvănescu, Diana-Gabriela Ilaș, Cosmin Cîtu, Corina Vernic, Ioan Sas

**Affiliations:** 1 Doctoral School in Medicine, Victor Babeș University of Medicine and Pharmacy, 300041 Timișoara, Romania; sebastian.ciurescu@umft.ro (S.C.); borozan.florina@umft.ro (F.B.); raluca.reitmayer@umft.ro (R.P.); 2 Department of Obstetrics and Gynecology, Victor Babeş University of Medicine and Pharmacy, 300041 Timișoara, Romania; citu.ioan@umft.ro (C.C.); sas.ioan@umft.ro (I.S.); 3 Department XV of Orthopaedics, Traumatology, Urology and Medical Imaging, Discipline of Radiology and Medical Imaging, Victor Babeș University of Medicine and Pharmacy, 300041 Timișoara, Romania; cerbu.simona@umft.ro; 4 Division of Cardiovascular Surgery, Department VI Cardiology, Victor Babeș University of Medicine and Pharmacy, 300041 Timișoara, Romania; dima.ciprian@umft.ro; 5 Department of Medical Semiology, Victor Babeș University of Medicine and Pharmacy, 300041 Timișoara, Romania; diana_ilas@yahoo.com; 6 Department III—Functional Science, Discipline of Medical Informatics and Biostatistics, Victor Babeș University of Medicine and Pharmacy, 300041 Timișoara, Romania

**Keywords:** AI-assisted breast cancer detection, medical image processing

## Abstract

*Background and Objectives*: Breast cancer is a leading global health challenge, where early detection is essential for improving survival outcomes. Two-dimensional (2D) mammography is the established standard for breast cancer screening; however, its diagnostic accuracy is limited by factors such as breast density and inter-reader variability. Recent advances in artificial intelligence (AI) have shown promise in enhancing radiological interpretation. This study aimed to assess the utility of AI in improving lesion detection and classification in 2D mammography. *Materials and Methods*: A retrospective analysis was performed on a dataset of 578 mammographic images obtained from a single radiology center. The dataset consisted of 36% pathologic and 64% normal cases, and was partitioned into training (403 images), validation (87 images), and test (88 images) sets. Image preprocessing involved grayscale conversion, contrast-limited adaptive histogram equalization (CLAHE), noise reduction, and sharpening. A convolutional neural network (CNN) model was developed using transfer learning with ResNet50. Model performance was evaluated using sensitivity, specificity, accuracy, and area under the receiver operating characteristic (AUC-ROC) curve. *Results*: The AI model achieved an overall classification accuracy of 88.5% and an AUC-ROC of 0.93, demonstrating strong discriminative capability between normal and pathologic cases. Notably, the model exhibited a high specificity of 92.7%, contributing to a reduction in false positives and improved screening efficiency. *Conclusions*: AI-assisted 2D mammography holds potential to enhance breast cancer detection by improving lesion classification and reducing false-positive findings. Although the model achieved high specificity, further optimization is required to minimize false negatives. Future efforts should aim to improve model sensitivity, incorporate multimodal imaging techniques, and validate results across larger, multicenter prospective cohorts to ensure effective integration into clinical radiology workflows.

## 1. Introduction

Breast cancer remains a significant global health concern, ranking as the most frequently diagnosed cancer among women and a leading cause of cancer-related mortality. In 2020, it was estimated that over 2.3 million new cases were diagnosed worldwide, accounting for approximately 11.7% of all cancer cases [1]. The disease burden varies significantly across different regions, with higher mortality rates observed in low- and middle-income countries due to limited access to early detection and advanced treatment modalities [2]. Early detection through organized screening programs has proven to be a crucial factor in improving patient outcomes and reducing mortality rates. However, despite significant advancements in screening technologies, limitations persist in terms of diagnostic accuracy and the potential for overdiagnosis and false positives [3].

Among the available imaging modalities, two-dimensional (2D) mammography remains the gold standard for routine breast cancer screening due to its widespread availability, cost-effectiveness, and proven diagnostic utility [4,5]. While digital mammography has improved image quality and diagnostic precision compared to film-based mammography, the inherent limitations of 2D imaging persist, particularly in women with dense breast tissue, where sensitivity is significantly reduced [6,7]. Breast density not only compromises the ability to detect malignancies but also serves as an independent risk factor for breast cancer, further complicating the screening process [8,9,10]. These limitations underscore the pressing need for advanced computational tools capable of enhancing diagnostic accuracy. AI, particularly deep learning models such as convolutional neural networks (CNNs), offers a promising solution by autonomously learning to detect complex imaging patterns. By leveraging vast datasets, AI models can reduce human subjectivity, mitigate inter-reader variability, and improve lesion detection in cases where breast tissue density reduces visibility. The ability of AI to continuously refine its predictive capabilities based on radiological feedback makes it an attractive adjunct to traditional screening methods. Additionally, reader-to-reader variability among radiologists may result in diagnostic inconsistencies, necessitating supplementary imaging techniques such as ultrasound or magnetic resonance imaging (MRI) for more accurate lesion characterization [8,11].

Recent advancements in artificial intelligence have demonstrated significant potential in augmenting radiological interpretation and improving the accuracy of breast cancer detection [12,13,14]. AI-driven algorithms, particularly deep learning models based on convolutional neural networks (CNNs), have shown remarkable performance in analyzing mammographic images, identifying subtle abnormalities, and reducing false-positive and false-negative rates [4,15]. CNNs are well-suited for image processing tasks in medical imaging, including mammography [15]. Unlike traditional machine learning approaches that require manual feature extraction, CNNs autonomously learn hierarchical representations of image features through stacked layers of convolutional and pooling operations. The initial layers capture low-level patterns, such as edges and textures, while deeper layers extract more complex structures, allowing the network to differentiate between normal and pathological findings [6,13]. CNNs have demonstrated superior performance in medical imaging by leveraging large annotated datasets and continuously refining their detection capabilities [4,16]. Several studies have validated their ability to achieve radiologist-level accuracy in identifying breast cancer, reducing inter-reader variability, and minimizing diagnostic errors [8,12,17]. These advancements underscore the growing role of deep learning in improving breast cancer detection and streamlining mammography-based screening processes. Several studies have indicated that AI can match or even surpass human experts in detecting breast cancer in mammographic images, thereby standardizing interpretations and mitigating radiologist fatigue [6,18]. Moreover, AI-driven computer-aided detection (CAD) systems have been increasingly integrated into clinical workflows, assisting radiologists in identifying suspicious findings with greater efficiency and accuracy [19]. The ability of AI to learn from vast datasets and continuously improve its predictive capabilities holds promise for revolutionizing breast cancer screening and diagnosis.

Despite the growing body of evidence supporting AI-assisted mammography, challenges remain regarding its widespread clinical implementation. Concerns related to data privacy, model generalizability, and algorithm transparency necessitate further investigation before AI can be fully integrated into routine clinical practice [18,20]. Additionally, the ethical implications of AI-assisted diagnosis, particularly regarding accountability and decision-making autonomy, must be carefully considered to ensure patient safety and trust in AI-driven healthcare solutions [21].

This study aims to explore the utility of AI in the interpretation of 2D mammographic images, focusing on improving lesion detection and classification accuracy. By leveraging deep learning methodologies, we seek to assess the performance of AI-assisted screening in comparison to traditional radiological assessment. Additionally, we will evaluate the clinical feasibility, limitations, and future prospects of integrating AI-driven tools into routine breast cancer screening protocols. The expected outcome of this research is to provide a validated AI model capable of enhancing breast cancer detection, ultimately improving patient outcomes through more accurate and efficient screening strategies.

This paper begins with a review of existing AI methodologies applied in breast cancer detection using mammography. The materials and methods section details dataset characteristics, preprocessing techniques, and AI model development. The results highlight model performance metrics, including accuracy, sensitivity, specificity, and AUC-ROC. The discussion compares AI-assisted detection with traditional radiological interpretation, analyzing clinical feasibility, limitations, and areas for improvement. Finally, the conclusion summarizes key findings and explores future research directions for integrating AI into breast cancer screening workflows.

The integration of artificial intelligence in breast cancer detection has been extensively explored in recent years, leading to the development of various machine learning and deep learning approaches. These techniques aim to enhance lesion detection and classification accuracy in mammography while addressing challenges such as inter-reader variability and the limitations of traditional screening methods. This section provides an overview of previous research in the field, focusing on machine learning-based approaches, deep learning methods, and ensemble learning techniques.

### 1.1. Machine Learning-Based Approaches for Breast Cancer Detection

Traditional machine learning techniques have been widely used in mammographic image analysis, relying on handcrafted feature extraction to classify lesions. Methods such as support vector machines (SVMs), random forests, and k-nearest neighbors have demonstrated the ability to differentiate between benign and malignant findings based on manually selected radiomic features, including texture, shape, and intensity [22]. While these approaches have shown reasonable classification performance, their effectiveness is often constrained by the quality of feature engineering and the inability to generalize across diverse mammographic datasets [23]. Furthermore, these methods typically require an expert-driven selection of relevant features, which can introduce bias and limit adaptability to complex imaging variations. Despite their limitations, traditional machine learning models have laid the foundation for the transition to more advanced deep learning techniques, which offer automated feature extraction and improved classification capabilities [4].

### 1.2. Deep Learning Methods for Mammography Analysis

The advent of deep learning has significantly improved breast cancer detection in mammography by allowing models to learn hierarchical representations of imaging data without the need for manual feature engineering [24]. CNNs have emerged as the predominant approach, demonstrating superior performance in identifying malignant lesions compared to traditional machine learning classifiers [17]. By leveraging multi-layer architectures, CNNs can capture intricate spatial features within breast tissue, thereby improving lesion characterization. The implementation of transfer learning has further enhanced CNN-based mammographic analysis, with models such as ResNet and VGG [13]. However, despite their advantages, deep learning models face challenges related to interpretability and generalization. The presence of false positives and false negatives remains a concern, as AI-based screening tools must align with the high sensitivity and specificity standards required for clinical adoption [25]. Addressing these challenges requires ongoing refinements in model training strategies, data augmentation techniques, and the integration of multimodal imaging data [26].

### 1.3. Ensemble Learning Techniques for Breast Cancer Classification

To mitigate the limitations of single deep learning models, ensemble learning techniques have been explored as a means of improving diagnostic accuracy and robustness in breast cancer detection [27]. By combining multiple classifiers, ensemble methods such as hybrid CNN–decision tree models, stacking architectures, and majority voting strategies have demonstrated enhanced reliability in distinguishing between normal and pathologic cases [28]. Studies have shown that integrating CNN-based feature extraction with ensemble classifiers such as XGBoost and AdaBoost can improve sensitivity while maintaining high specificity [29]. Additionally, ensemble learning can help address the issue of class imbalance by leveraging multiple predictive models that complement each other’s strengths [10]. Recent developments have also incorporated synthetic data generation through generative adversarial networks (GANs) to bolster ensemble model performance. For instance, Amritanjali et al. [30] used a Pix2Pix GAN framework to augment MRI-based brain tumor datasets, enhancing classification in rare and underrepresented classes. Similarly, Durur-Subasi and Özçelik [31] demonstrated that combining conditional deep convolutional GAN-generated datasets with ensemble architectures significantly improved diagnostic reliability in brain tumor classification. While these advanced data augmentation strategies have primarily been applied to neurological imaging, their underlying principles hold substantial potential for improving ensemble-based breast cancer detection. Further research is needed to optimize these frameworks and validate their performance in large-scale, multimodal clinical settings.

## 2. Materials and Methods

### 2.1. Study Design and Ethical Approval

This retrospective study aimed to evaluate the effectiveness of an AI-based approach for detecting breast pathologies in 2D mammographic images. The dataset was sourced from the Radiology Department of the Municipality Emergency Clinical Hospital Timisoara between 2024 and January 2025. The initial dataset comprised 750 mammograms, capturing a diverse demographic distribution. To ensure robustness, the dataset included mammograms from patients across multiple age groups (35–80 years old), encompassing both dense and fatty breast tissues. Each mammogram was categorized according to BI-RADS classifications (1–5), ensuring the inclusion of diagnostically challenging cases such as heterogeneously dense breast patterns and subtle microcalcifications. Ethical approval was granted by the Institutional Review Board of the University of Medicine and Pharmacy Victor Babes Timisoara and the Municipality Emergency Clinical Hospital Timisoara (protocol number 43/20 October 2023). This study complied with the principles outlined in the Declaration of Helsinki.

### 2.2. Patient Population and Imaging Dataset

The dataset used in this study was obtained from the Radiology Department of the Municipal Emergency Clinical Hospital Timișoara between 2024 and January 2025. Mammographic images were acquired using standard full-field digital mammography (FFDM) units, capturing both cranio-caudal (CC) and mediolateral oblique (MLO) views. The dataset includes both normal and pathological cases, ensuring a representative sample for training an AI-assisted classification model.

**Figure 1:**
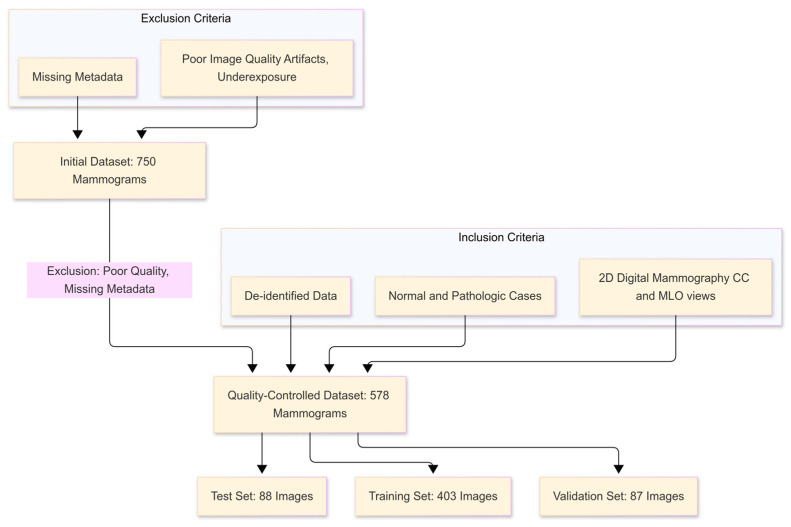
This flowchart summarizes the dataset selection process. From 750 initial images, 578 high-quality, de-identified mammograms were retained. Each image represents a single view (CC or MLO) and was treated independently during training. The dataset was divided into training (403), validation (87), and test (88) sets to support robust model development and evaluation.

To maintain high-quality data, a rigorous selection process was applied. As depicted in Figure 1, exclusion criteria included images with poor quality, such as motion artifacts, underexposure, or missing patient metadata. Following this quality control step, a total of 578 images from 100 participants were selected for analysis. These images were divided into three subsets: 403 images for training, 87 for validation, and 88 for testing. Each mammogram corresponds to a single view (either cranio-caudal or mediolateral oblique), rather than paired images. Both views were included independently in the dataset and treated as distinct input instances during training and evaluation. This structured partitioning allowed for robust model training and performance evaluation. Prior to analysis, all personal identifiers were removed to ensure compliance with ethical guidelines, including HIPAA and GDPR, safeguarding patient confidentiality.

While publicly available mammography datasets such as DDSM, CBIS-DDSM, MIAS, and INbreast are frequently used in breast cancer research, our dataset offers significant advantages. Unlike heterogeneous public datasets, which often contain images from multiple institutions with varying acquisition parameters, this dataset was collected under standardized imaging conditions, ensuring uniform contrast, resolution, and exposure levels. This consistency minimizes technical variability, allowing the AI model to focus on distinguishing pathological features rather than adapting to differing imaging conditions.

Another key advantage is the clinical relevance of the dataset. Unlike many publicly available datasets, which may contain outdated imaging protocols or lack biopsy confirmation, this dataset was expert-annotated by certified radiologists and includes BI-RADS classifications ranging from 1 to 5, with confirmed malignancies. Additionally, it includes diagnostically challenging cases, such as dense breast tissue and subtle microcalcifications, which are often underrepresented in publicly available databases but are critical for developing AI models applicable to real-world screening settings.

### 2.3. Image Preprocessing

To enhance the diagnostic quality of mammographic images and optimize model performance, a structured preprocessing pipeline was implemented. This process was designed to standardize the dataset, improve image contrast, and minimize artifacts that could interfere with lesion detection. The first step involved converting all images to 8-bit grayscale, a necessary transformation to ensure consistency across the dataset while preserving fine structural details. This step also facilitated compatibility with deep learning models, as grayscale images reduce computational complexity without compromising essential mammographic features.

Following grayscale conversion, data normalization was applied to stabilize pixel intensity values across all images. Each pixel was scaled to a range between 0 and 1 by dividing its value by 255.0, ensuring uniform intensity distribution throughout the dataset. This normalization step was critical in preventing numerical instability during training and enabling the model to generalize effectively across images captured under varying exposure conditions.

To enhance the visibility of subtle lesions, contrast-limited adaptive histogram equalization (CLAHE) was utilized. This technique improved local contrast while mitigating the risk of overamplifying noise, a crucial factor in detecting key radiological features such as microcalcifications and architectural distortions commonly associated with malignancies. In addition to contrast enhancement, noise reduction was performed using a combination of bilateral filtering and median filtering. The bilateral filter effectively removed high-frequency noise while preserving edges, whereas the median filter eliminated speckle noise, a frequent occurrence in digital mammography. This noise reduction approach helped ensure that fine anatomical structures remained intact while non-informative variations were suppressed.

Given the relatively small size of the dataset, data augmentation techniques were employed to improve generalization and prevent overfitting. Images were horizontally flipped to simulate variations in breast positioning during screening. Additionally, rotational transformations of 90°, 180°, and 270° were applied to account for different imaging orientations. Intensity variations were also introduced to replicate real-world exposure conditions and lighting differences, ensuring that the model could adapt to varying image acquisition environments.

To further enhance lesion detectability, edge sharpening was applied using a Laplacian filter. This step was essential for improving the delineation of tumor margins, microcalcifications, and architectural distortions, all of which are critical features for the AI model in distinguishing normal from pathological cases. By implementing these preprocessing techniques, the dataset was refined to provide high-quality input for the deep learning models, ensuring optimal conditions for feature extraction and classification.

### 2.4. CNN and ResNet50 Model Architecture

To classify mammographic images into normal and pathological categories, two deep learning architectures were developed and evaluated. The first approach involved training a custom convolutional neural network from scratch, while the second utilized transfer learning with the ResNet50 architecture to leverage pre-trained feature extraction capabilities.

The custom CNN model consisted of three convolutional layers, each equipped with 3 × 3 kernels and ReLU activation functions to introduce non-linearity. These convolutional layers were followed by max pooling layers, which downsampled the feature maps while retaining the most salient features. The extracted features were then passed through a fully connected layer with 128 neurons, which aggregated the learned patterns before final classification. A softmax output layer was used to distinguish between normal and pathological cases, ensuring that the model produced a probability distribution for each classification decision.

In addition to the CNN model, a more advanced approach was implemented using ResNet50, a deep residual network pre-trained on ImageNet. Transfer learning was employed to fine-tune the model for mammographic classification, leveraging its robust feature extraction capabilities. The final layers of ResNet50 were modified to better suit the specific task at hand. A global average pooling layer replaced the dense fully connected layers, followed by a 128-neuron dense layer with ReLU activation and a softmax output layer for binary classification. Given that ResNet50 expects three-channel input images, the grayscale mammograms were replicated across three channels to maintain compatibility. Initially, the pre-trained layers of ResNet50 were frozen, allowing only the newly added layers to be trained. Subsequently, selective fine-tuning of deeper layers was applied to further optimize feature extraction for mammographic analysis.

#### Justification for Transfer Learning

The decision to implement transfer learning was based on the relatively limited size of the dataset, which posed a risk of overfitting if a deep CNN were trained from scratch. ResNet50, pre-trained on millions of natural images, provided a powerful feature extraction framework that could be fine-tuned to identify relevant mammographic structures. This approach offered several advantages.

First, faster convergence was achieved, as the model was initialized with learned features, reducing the number of epochs required to reach optimal performance. Second, improved generalization was observed since transfer learning allowed the model to leverage deep spatial feature representations, making it more robust to variations in imaging conditions. Lastly, computational efficiency was significantly improved, as training a deep CNN from scratch is resource-intensive. By utilizing a pre-trained model, training time and hardware requirements were substantially reduced, making the approach more practical for large-scale implementation in clinical settings.

### 2.5. Training, Validation, and Model Selection

For consistent model evaluation, the dataset was stratified into training, validation, and test sets. Specifically, 70% of the data was allocated for training, 15% for validation, and 15% for testing. Throughout training, model selection was guided by performance metrics and optimization techniques to prevent overfitting and maximize classification accuracy.

Early stopping was implemented to monitor validation loss, halting training if no improvement was observed for five consecutive epochs. Learning rate reduction was also applied, adjusting the learning rate dynamically by halving it after three epochs of stagnation in validation performance. The models were trained using a batch size of 32, which provided a balance between computational efficiency and gradient stability. Training was conducted for a maximum of 20 epochs, with performance monitored based on accuracy, loss, sensitivity, specificity, and AUC-ROC (Area Under the Receiver Operating Characteristic Curve) to determine the best-performing model. To further optimize model performance, hyperparameter tuning was conducted using a grid search approach to identify the most effective dropout rate, batch size, and learning rate schedule. The final configuration was determined based on validation accuracy and loss trends, selecting a dropout rate of 0.5, a batch size of 32, and an adaptive learning rate decay applied after three epochs of stagnation in validation performance. An early stopping criterion with a patience of five epochs was used to halt training if validation loss ceased to improve, preventing overfitting. The Adam optimizer was employed with an initial learning rate of 0.001, which was dynamically reduced by a factor of 0.5 when validation loss plateaued for three consecutive epochs. To ensure robust generalization, L2 weight decay regularization was explored but was found to have minimal impact compared to dropout-based tuning. The best-performing model was selected based on validation set accuracy, sensitivity, and specificity, with final testing conducted on the held-out test set. To further interpret AI decision-making, grad-CAM (gradient-weighted class activation mapping) visualization was used to assess whether the model’s focus aligned with radiologist-marked regions of interest. The final trained model was saved in HDF5 format to facilitate reproducibility and further clinical validation.

### 2.6. Model Evaluation on the Test Set

All preprocessing, training, and evaluation procedures were implemented using Python 3.10. The deep learning models were developed with TensorFlow v2.12.0 and Keras API v2.12.0, while OpenCV v4.7.0.72 was used for image processing and scikit-learn for performance evaluation metrics and plotting tools. Performance assessment involved multiple analytical techniques to comprehensively evaluate the model’s diagnostic accuracy. To quantify the effectiveness of the AI model, standard performance metrics were computed. Accuracy was measured as the proportion of correctly classified mammograms. Sensitivity (recall) reflected the model’s ability to correctly detect pathological cases, ensuring that malignant lesions were not overlooked. Specificity was used to assess the model’s capacity to correctly classify normal cases while minimizing false positives. The F1-score, a harmonic mean of precision and recall, provided a balanced measure of the model’s ability to handle both false positives and false negatives. Finally, AUC-ROC was calculated to assess the model’s overall discriminative power, indicating how well it could separate normal and pathological cases. A confusion matrix analysis was conducted to differentiate between true positives, true negatives, false positives, and false negatives, providing deeper insights into the model’s classification behavior. Statistical comparisons were conducted using McNemar’s test to evaluate differences in paired binary classifications across models applied to the same set of 88 mammograms. This non-parametric method is appropriate for assessing the significance of discordant predictions in matched samples. The test was employed to compare the ResNet50 model with both EfficientNetB0 and VGG16, as well as with a radiologist. Additionally, a post-hoc power analysis was performed to determine whether the sample size was sufficient to detect the observed difference in specificity, using a two-sided test with a significance threshold of α = 0.05.

### 2.7. Model Optimization and Loss Function

To ensure robust training and minimize classification errors, the categorical cross-entropy loss function was employed, a widely used method for optimizing probability-based classification outputs in binary classification tasks. Gradient clipping was applied to prevent exploding gradients, ensuring numerical consistency throughout the learning process. Additionally, batch normalization was incorporated into the architecture to stabilize activation distributions and improve generalization by reducing internal covariate shifts. These optimization techniques were crucial in refining the model’s predictive accuracy and ensuring its robustness across different imaging conditions.

## 3. Results

### 3.1. Image Processing and Optimization for AI Training

**Figure 2:**
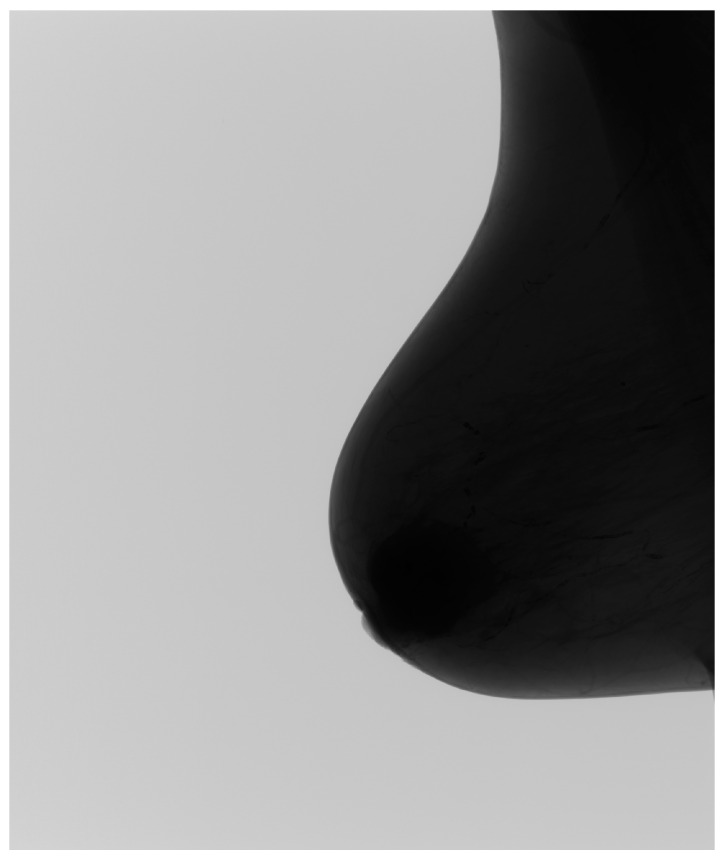
Example of DICOM to PNG conversion, demonstrating the preservation of essential mammographic structures.

The preprocessing of mammographic images plays a fundamental role in ensuring the effectiveness of AI-based classification. To create a dataset suitable for deep learning, images originally acquired in the Digital Imaging and Communications in Medicine (DICOM) format were converted into the Portable Network Graphics (PNG) format. This step ensured compatibility with modern deep learning frameworks while maintaining structural fidelity and preserving critical radiological details. Figure 2 illustrates this transformation, highlighting how the conversion process retains fine-scale features essential for lesion detection.

**Figure 3:**
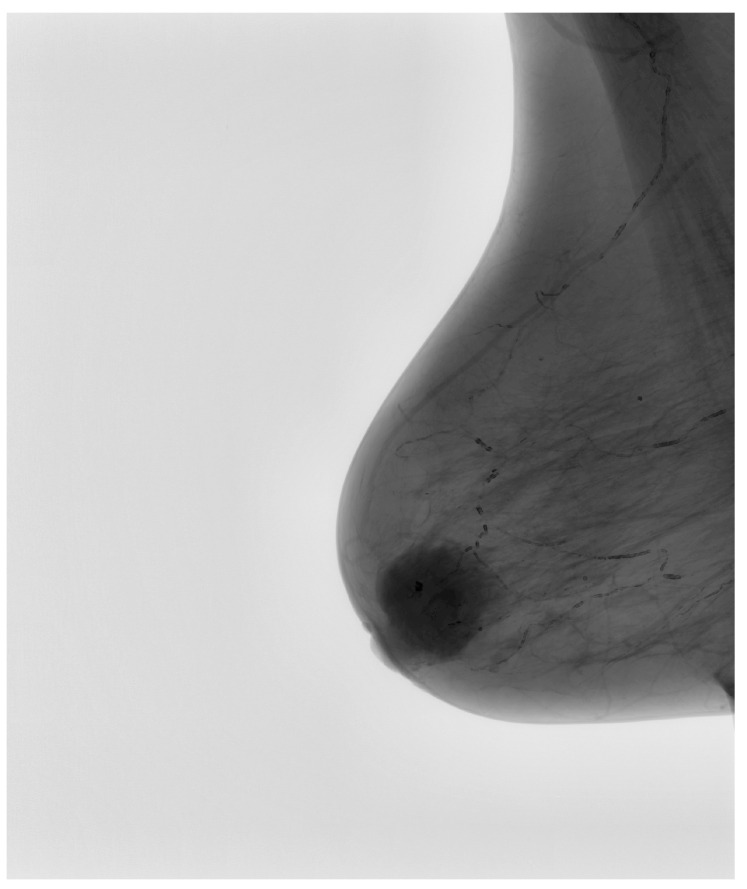
Intermediary-processed mammographic image showcasing enhanced contrast and denoising.

Following this conversion, a series of image enhancement techniques were applied to optimize the quality of mammograms before they were introduced into the convolutional neural network. Given that mammographic images inherently exhibit low contrast, particularly in cases involving dense breast tissue, contrast-limited adaptive histogram equalization was employed to improve the visibility of structural variations within the breast parenchyma. This enhancement method, as depicted in Figure 3, demonstrated a substantial improvement in contrast while simultaneously preventing overamplification of noise, which could otherwise introduce false-positive findings during AI-based classification.

In addition to contrast enhancement, noise reduction was performed using an advanced denoising algorithm to eliminate non-informative variations caused by acquisition artifacts or background irregularities. Reducing noise is particularly critical in mammographic AI applications, as unwanted fluctuations in pixel intensity can lead to the misinterpretation of tissue textures and edge structures. By carefully suppressing irrelevant noise while preserving pathologically relevant signals, the algorithm enhanced the clarity of microcalcifications, spiculations, and soft-tissue masses. The intermediary processed image, as shown in Figure 3, illustrates this refinement.

**Figure 4:**
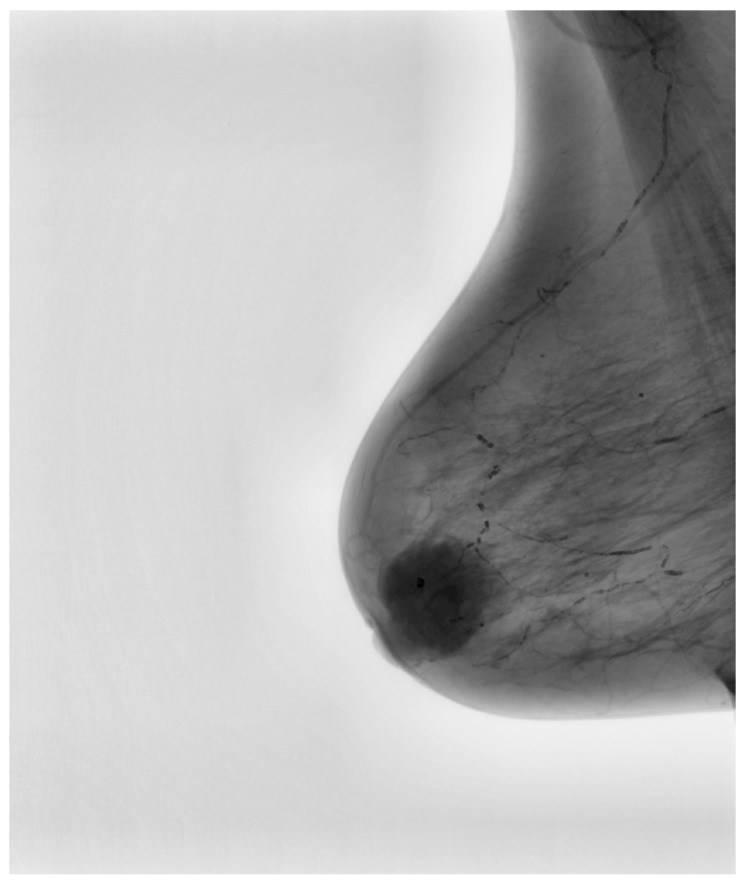
Final processed image, optimized for deep learning-based classification.

The final stage of preprocessing involved the application of image-sharpening techniques aimed at enhancing edge definition. Given that the appearance of suspicious lesions, including architectural distortions and microcalcifications, relies on their edge characteristics, sharpening the image facilitated better delineation of these features. Figure 4 demonstrates the final processed mammographic image optimized for subsequent AI training. The combination of CLAHE, noise reduction, and sharpening ensured that each image provided maximum diagnostic information while maintaining a standardized format, reducing heterogeneity that could otherwise compromise the learning process of the AI model.

### 3.2. AI Model Development, Training, and Performance Evaluation

#### 3.2.1. Training and Validation Performance

The AI model was developed and trained using a structured dataset consisting of 403 training images, 87 validation images, and 88 test images, ensuring a balanced distribution between normal and pathological cases. The deep learning architecture involved a custom CNN model for initial experiments, followed by a refined approach leveraging transfer learning with ResNet50, a robust CNN architecture pre-trained on large-scale image datasets.

During the training phase, the CNN model exhibited a gradual yet consistent increase in classification performance for 20 epochs. Training accuracy initially started at around 60%, demonstrated a steady increase, and ultimately approached nearly 100% by the final epochs. The validation accuracy followed a similar trajectory, beginning in the mid-60% range, experiencing minor fluctuations, and eventually stabilizing at 92–94% by the later stages of training. The presence of an initial drop in validation accuracy, followed by a steady increase, suggested that the model underwent an adjustment phase before learning to generalize effectively to previously unseen data.

**Figure 5:**
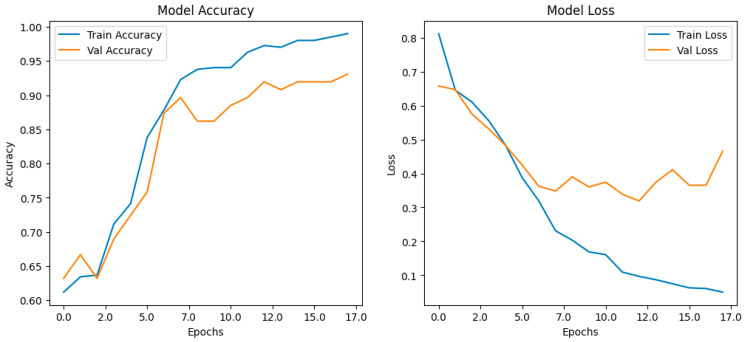
Training and validation accuracy curves after fine-tuning, showing performance improvements across epochs. The graph illustrates the model’s learning progression, with both training and validation accuracy increasing steadily over successive epochs. The plateau in validation accuracy towards the final epochs indicates model stabilization and reduced overfitting, confirming generalization to unseen data.

The AI Model Performance post-fine-tuning, presented in Figure 5, provides a comprehensive visualization of the training and validation accuracy curves, illustrating the steady improvement in classification performance across multiple epochs. The graph highlights the increasing separation between the training and validation curves, indicative of the model’s progression toward optimized feature extraction. The observed plateau in validation accuracy toward the final epochs suggests that the model achieved a stable generalization level, reinforcing its robustness when applied to unseen mammographic data.

The loss function analysis revealed further insights into the model’s learning dynamics. During the early epochs, the training loss exhibited a sharp decline from an initial value of from 0.8 to below 0.1, demonstrating a rapid optimization process. The validation loss, while following a decreasing trend, plateaued at a value between 0.3 and 0.4, with a minor upward fluctuation in the final epochs. This divergence between training and validation loss suggested that while the model learned highly detailed representations from the training data, it also demonstrated mild overfitting, a common occurrence in deep learning models when exposed to limited datasets. However, given that validation accuracy remained consistently high, the degree of overfitting was not deemed excessive.

#### 3.2.2. Model Performance on the Test Set

The generalization capability of the AI models was assessed using a held-out test set comprising 88 mammograms. The classification results were quantified using standard performance metrics, including accuracy, sensitivity, specificity, and AUC-ROC. The models tested included a custom ResNet50-based AI model, VGG16, and EfficientNetB0. Their results were compared with radiologists’ diagnostic accuracy in conventional 2D mammography.

A summary of the model performances is presented in Table 1, providing a comparative view of their classification capabilities:

The ResNet50-based AI model demonstrated statistically superior classification performance compared to all evaluated alternatives. McNemar’s test indicated significant differences in prediction outcomes relative to EfficientNetB0 (*p* < 0.01) and VGG16 (*p* < 0.001), the latter failing to correctly identify any pathological cases. When benchmarked against the radiologist, ResNet50 exhibited a significantly lower rate of discordant classifications (*p* = 0.049), largely attributable to a reduction in false positives, which contributed to its higher specificity (92.7% vs. 77%). A post-hoc power analysis confirmed that the sample size (n = 88) was sufficient to detect this effect, yielding a statistical power of 87%. These findings affirm the comparative robustness of the proposed model over both conventional CNN architectures and expert human assessment.

In terms of discriminative performance, ResNet50 achieved an AUC-ROC of 0.93 and an overall test accuracy of 88.5%, with a sensitivity of 81% and a notably high specificity of 92.7%. This profile indicates reliable differentiation between normal and pathological cases and demonstrates improved diagnostic precision compared to traditional radiological interpretation. By contrast, VGG16 was clinically non-viable, with a sensitivity of 0% and a total accuracy of only 63%. Despite an AUC-ROC of 0.91, the EfficientNetB0 model showed suboptimal sensitivity (25%), failing to detect the majority of malignant cases, thereby limiting its diagnostic utility.

These findings suggest that AI-based screening has the potential to complement radiological assessments by reducing false positives while maintaining clinically acceptable sensitivity levels. The AI models demonstrated particular strength in specificity, a key factor in minimizing unnecessary follow-ups and biopsies, thus improving the efficiency of breast cancer screening programs.

**Figure 6:**
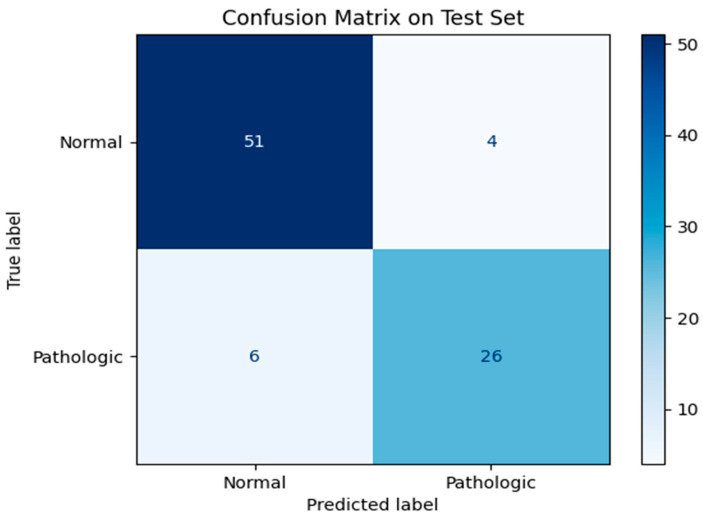
Confusion matrix illustrating the classification outcomes on the test set.

A detailed confusion matrix analysis (Figure 6) highlighted the classification strengths and limitations of the ResNet50-based proposed AI model. The model correctly identified 26 true positive cases and 51 true negative cases while misclassifying 6 false negative cases and 4 false positive cases. Despite achieving a high specificity of 92.7%, the presence of false negatives suggests areas for improvement, particularly in reducing missed malignancies.

**Figure 7:**
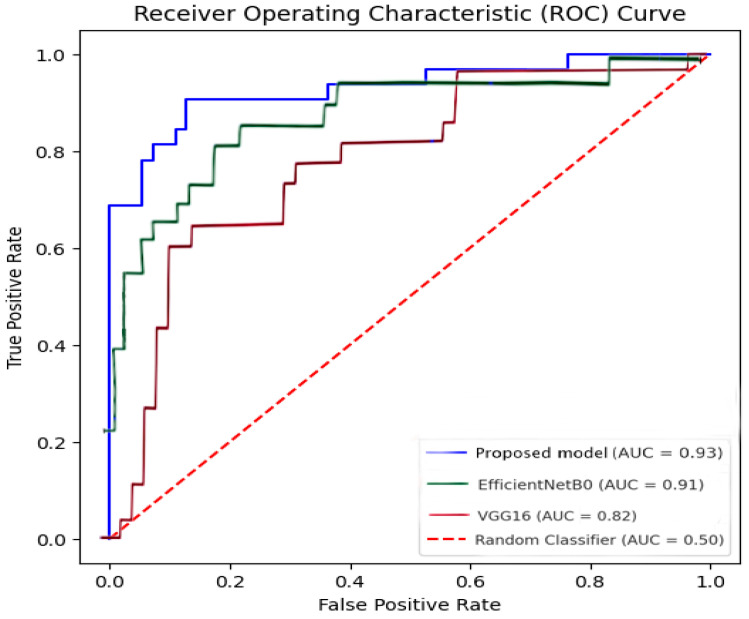
Unified ROC curve comparison of AI models in 2D mammography classification. The graph illustrates the performance of different AI models (proposed model, EfficientNetB0, and VGG16) in distinguishing between normal and pathological mammographic cases. The AUC reflects each model’s discriminative power, with the proposed model achieving the highest AUC (0.93), indicating superior classification performance. The diagonal dashed line represents a random classifier’s baseline performance (AUC = 0.50).

The unified ROC curve (Figure 7) provides a visual representation of the classification performance of different AI models compared to radiologists. This allows for a direct assessment of each model’s discriminative power and highlights the superior performance of our fine-tuned model in distinguishing between normal and pathological cases.

## 4. Discussion

Integrating artificial intelligence into 2D mammography continues to attract significant attention for its potential to improve both the accuracy and efficiency of breast cancer screening, especially when large-scale programs must manage substantial imaging volumes. By comparing our present findings with earlier investigations, it becomes apparent that an AI-augmented workflow can provide consistent gains in lesion detection rates and reductions in false-positive callbacks, a trend consistent with observations in other diagnostic imaging applications [32]. Recent research has further emphasized the potential of AI-driven approaches in improving diagnostic workflows, particularly in medical imaging, by refining detection accuracy and minimizing diagnostic errors [33]. Recent studies evaluating AI models for mammographic analysis have reported sensitivity ranging from 80 to 85% and specificity between 78 and 89%. Compared to these benchmarks, our model’s sensitivity of 81% aligns with prior findings, while its higher specificity (92.7%) suggests superior differentiation between benign and malignant cases. However, our model exhibited six false negatives, a concern that other AI studies have addressed through ensemble learning or integrating AI as a second reader alongside radiologists. By leveraging hybrid AI models or adjusting decision thresholds, sensitivity could be further enhanced while maintaining clinically acceptable specificity levels. These enhancements are grounded in the capacity of deep learning models to extract and synthesize features that may escape human observers during single or double reading, an observation that is supported by previous studies involving breast imaging radiologists [34]. Improving detection consistency is particularly relevant in regions where radiologist shortages and imaging backlogs persist, as high throughput often amplifies the need for efficient, standardized interpretations [35,36].

The test dataset exhibited a moderate class imbalance, with 36% pathological and 64% normal cases (a 1:1.75 ratio), which can potentially affect sensitivity, specificity, and overall classification behavior. To mitigate the impact of this imbalance, we supplemented traditional performance measures with more robust metrics, such as AUC-ROC and the F1-score, both of which provide a more reliable assessment when class distributions are unequal. The model’s high AUC-ROC (0.93) and balanced sensitivity/specificity suggest that performance was not disproportionately driven by the dominant class. Nevertheless, we acknowledge that even moderate imbalance may bias classification thresholds, and future work will explore training strategies involving class weighting, resampling, or stratified cross-validation.

Although the dataset was collected under standardized conditions at a single institution, external validation using public datasets such as CBIS-DDSM or INbreast remains essential to confirm the generalizability of the model. This remains a key objective in our future research agenda.

An analysis of the six false-negative cases revealed that most occurred in the context of dense breast tissue, where lesions presented low contrast and lacked microcalcifications or architectural distortion. These radiographic features likely contributed to missed detections. These findings highlight the need for further optimization of the model’s sensitivity to subtle patterns, particularly in mammographically dense breasts, and support its use as an adjunctive rather than standalone tool.

Beyond conventional screening applications, AI models such as the one proposed here may assist in more complex diagnostic scenarios, including cases of carcinoma of unknown primary (CUP). In such instances, the breast represents a frequent but often occult source, where AI-assisted interpretation could improve diagnostic confidence. This perspective is supported by recent literature on integrating AI into ambiguous clinical contexts [37].

Although radiologists’ expertise remains essential, an AI system can offset the subjective elements of screening through its ability to learn complex patterns from large datasets. In line with this approach, [29] recent studies demonstrated how deep super-resolution combined with convolutional neural networks can enhance sensitivity to subtle pathologic signs like ductal carcinoma in situ (DCIS). This lesion frequently poses challenges in traditional screening protocols. Studies [38] have shown that AI-based systems can support radiologists in identifying early breast lesions, translating into improved tumor detection rates. Furthermore, Aswiga et al. [39] underscored the versatility of AI by illustrating its ability to more accurately classify microcalcifications across diverse imaging conditions. An essential advantage observed in our findings, consistent with these broader investigations, concerns specificity and its effect on recall rates, an outcome that can be clinically and psychologically significant for patients. False-positive readings routinely prompt additional tests and can increase patient anxiety, thereby complicating follow-up logistics; Raiaan et al. [40] highlighted the practical and economic implications of these excessive callbacks, underscoring the value of any measures, such as AI triaging, that can mitigate them.

Technological advancements in multimodal imaging have also begun to interface with AI-augmented 2D mammography to form hybrid protocols that may yield even higher detection accuracy. Adapa et al. [41] presented initial results that integrated ultrasound data, revealing improved characterization of suspicious lesions, especially in patients with dense breast tissue. The interpretability of AI decisions is another concern integral to widespread clinical adoption. Amritanjali et al. [42] applied layer-wise relevance propagation to illustrate how CNNs identify critical image regions. Their results suggest that transparent networks may not only refine radiologists’ confidence in AI outputs but also enable more nuanced second-look evaluations in ambiguous or borderline presentations. Carierro et al. [43] discussed a continuous learning framework in which the model is routinely retrained on newly acquired data, thereby allowing updates to reflect changes in imaging technology, demographics, and local disease prevalence. The need to maintain patient confidentiality while benefiting from large, decentralized datasets has promoted research into federated learning approaches; Durur-Subasi et al. [44] documented how combining local model updates from multiple institutions can preserve privacy while bolstering generalizability and performance.

Future perspectives in AI-augmented 2D mammography lie at the intersection of clinical feasibility, algorithmic refinement, and health policy considerations. Future validation studies should explore the integration of hybrid AI models into real-world breast cancer screening programs. Prospective multi-center studies with diverse patient populations would be essential to assess the generalizability of these AI frameworks. Additionally, implementing real-time AI decision support tools could improve workflow efficiency by assisting radiologists in prioritizing high-risk cases for additional review. To ensure smooth clinical adoption, explainability techniques such as heatmaps, attention-based visualization, or layer-wise relevance propagation should be incorporated, allowing radiologists to interpret AI-generated predictions with greater confidence. Ultimately, by leveraging ensemble learning and multimodal AI architectures, breast cancer screening programs may significantly enhance early cancer detection, leading to improved patient outcomes and reduced diagnostic uncertainty. According to Hejduk et al. [45], prospective trials that measure AI’s effect not only on immediate diagnostic indicators but also on patient-centered outcomes are crucial for establishing how these technologies can be safely and effectively deployed. This aligns with the observations of Kundu et al. and Durur-Subasi et al. [44,46], who highlighted the regulatory and ethical challenges of AI-driven imaging, thereby emphasizing the need for a rigorous framework that ensures patient safety and public trust. An additional dimension is the implementation of practical quality assurance protocols, which Hejduk et al. [45] suggested may include systematic audits of AI predictions and real-time feedback loops that alert clinicians to potential misclassifications. Moreover, equitable AI deployment remains an urgent concern, as Kundu et al. [46] explored in their work on bias and data imbalance in underserved populations. Efforts to guarantee that AI-driven mammography benefits all demographic segments will need to address disparities in imaging availability, representativeness of training data, and varied healthcare infrastructure.

### 4.1. Limitations of This Study

Despite being promising, the transition to clinical usage demands a thorough account of limitations. From an infrastructural perspective, many facilities may be unprepared for the computational demands of high-parameter AI models. Even though cloud-based solutions partially lessen the requirement for on-site high-performance servers, considerations such as internet reliability and adherence to data privacy laws remain complex, as indicated by Wang [22]. A key challenge for AI deployment in real-world radiology workflows is interpretability. Many AI models function as black-box systems, making it difficult for radiologists to understand why a particular decision was made. Addressing this limitation requires the integration of attention-based visualization methods, such as layer-wise relevance propagation (LRP) or saliency maps, to highlight which regions of an image influenced AI classification decisions. This transparency could improve clinician trust and facilitate AI adoption in clinical practice. Furthermore, federated learning could address privacy concerns by allowing AI models to train across multiple institutions without data sharing, improving generalizability while preserving patient confidentiality.

In our study, despite favorable performance metrics, several limitations were observed. First, the model misclassified six pathological cases, primarily in dense breast tissue, where lesions exhibited poor contrast and subtle morphological characteristics. Second, training was constrained by the unavailability of high-performance GPUs, limiting the speed and efficiency of fine-tuning deep convolutional architectures. Similar observations were reported by Halling-Brown et al. [47], who emphasized the relevance of lightweight models in resource-constrained settings. Third, although expert annotations supported image labeling, the dataset originated from a single center, which may restrict generalizability to broader populations. Lastly, external validation was not conducted on large public datasets, such as INbreast or CBIS-DDSM, which would have further substantiated the model’s robustness. These limitations highlight the need for multi-institutional collaborations, privacy-preserving training methods like federated learning, and the development of extensive, high-quality image repositories.

The acquisition of such large-scale, annotated datasets remains a significant bottleneck. Al-Karawi et al. [48] studied the impact of different GPU architectures on deep learning mammography pipelines, illustrating the critical role of dataset diversity and size in achieving unbiased model performance. Manigrasso et al. [26], who developed a large multicenter repository for AI in breast imaging, demonstrated that such efforts often require sustained collaboration among researchers, clinicians, and technology providers.

### 4.2. Clinical Implications and Considerations

The findings of this study underscore the potential clinical applications of AI-assisted mammographic interpretation in breast cancer screening programs. The AI model demonstrated high specificity, reducing the likelihood of false positives, which, in turn, minimizes unnecessary patient anxiety, additional imaging, and biopsies. By significantly outperforming traditional mammographic specificity rates, the AI model offers a promising avenue for improving the efficiency of screening workflows while ensuring that normal cases are correctly identified with fewer erroneous recalls.

However, the presence of six false negative cases remains a clinical limitation, given that false negatives represent the most critical challenge in breast cancer screening. Missing malignant lesions in screening settings can result in delayed diagnosis and progression to more advanced stages of the disease, necessitating further refinement of the AI threshold to favor a higher sensitivity at the expense of a slight reduction in specificity. Hybrid approaches, combining AI-assisted diagnosis with radiologist review, could mitigate this limitation by providing a second layer of verification, particularly for cases where AI classification confidence is low.

With continued advancements in data augmentation, domain adaptation, and model interpretability, AI models such as the one developed in this study have the potential to serve as reliable adjunct tools, complementing radiologist expertise and enhancing diagnostic confidence in 2D mammographic screenings. Future validation studies should explore the integration of hybrid AI models into real-world breast cancer screening programs. Prospective multi-center studies with diverse patient populations would be essential to assess the generalizability of these AI frameworks. Additionally, implementing real-time AI decision support tools could improve workflow efficiency by assisting radiologists in prioritizing high-risk cases for additional review. To ensure smooth clinical adoption, explainability techniques such as heatmaps, attention-based visualization, or layer-wise relevance propagation should be incorporated, allowing radiologists to interpret AI-generated predictions with greater confidence. Ultimately, by leveraging ensemble learning and multimodal AI architectures, breast cancer screening programs may significantly enhance early cancer detection, leading to improved patient outcomes and reduced diagnostic uncertainty.

Taking all these elements into account, AI in 2D mammography has now reached a stage where it can alleviate some of the limitations of human-only interpretations while complementing the indispensable clinical judgment of radiologists. The reported improvements in sensitivity and specificity, along with the potential to reduce resource burden, underscore AI’s capacity to enhance early cancer detection and patient outcomes. Although the path to broader deployment involves addressing limited computational resources, refining continuous learning protocols, ensuring robust data governance, and reinforcing trust through explainable outputs, the momentum surrounding these innovations suggests that AI-based mammography is likely to gain a central position in global screening efforts. Through continued collaboration among radiologists, software engineers, data scientists, ethicists, and policymakers, the adoption of AI-driven mammography will become more seamless and equitable, ideally resulting in improved prevention, earlier interventions, and better overall prognoses for patients at risk of breast cancer.

## 5. Conclusions

This study highlights the potential of AI to enhance 2D mammography-based breast cancer screening. Our AI model, incorporating convolutional neural networks and transfer learning with ResNet50, demonstrated high accuracy (88.5%), strong discriminative power (AUC-ROC = 0.93), and improved specificity (92.7%) compared to traditional radiological interpretations. These results suggest that AI can enhance lesion detection, reduce false positives, and standardize radiological assessments.

Despite its promising performance, the model’s false negative rate (six missed cases) remains a clinical challenge, emphasizing the need for further refinements to enhance sensitivity. Future research should focus on improving model interpretability, integrating AI with multimodal imaging, and ensuring seamless clinical implementation.

In conclusion, AI has the potential to complement radiologists, improving diagnostic accuracy and workflow efficiency in breast cancer screening. While further validation through prospective clinical trials is necessary, AI-driven mammography represents a significant advancement toward more effective and accessible breast cancer detection.

Future research should focus on prospective validation of AI models in multi-center clinical settings, ensuring their generalizability across diverse patient populations. Additionally, integrating AI into radiology workflows as a decision-support tool rather than a standalone system may improve acceptance among clinicians. Lastly, enhancing AI interpretability through attention maps or decision transparency techniques will be critical in fostering trust and facilitating clinical implementation.

## Figures and Tables

**Table 1:** Performance comparison of AI models and radiologists in mammography classification.

Model	AUC-ROC	Accuracy (%)	Sensitivity (%)	Specificity (%)	*p*-Value
Proposed Model	0.93	88.5	81	92.7	-
EfficientNetB0	0.91	72	25	100	<0.01
VGG16	0.82	63	0	100	<0.001
Radiologists	0.80	80	82	77	0.049

## Data Availability

The raw data supporting the conclusions of this article will be made available by the authors on request.

## Abbreviations

The following abbreviations are used in this manuscript:

AI	Artificial Intelligence
AUC-ROC	Area Under the Receiver Operating Characteristic Curve
BI-RADS	Breast Imaging-Reporting and Data System
CAD	Computer-Aided Diagnosis
CC	Cranio-Caudal (mammographic view)
CNN	Convolutional Neural Network
CUP	Carcinoma of Unknown Primary
DCIS	Ductal Carcinoma In Situ
DICOM	Digital Imaging and Communications in Medicine
FFDM	Full-Field Digital Mammography
FN	False Negative
FP	False Positive
GAN	Generative Adversarial Network
Grad-CAM	Gradient-Weighted Class Activation Mapping
GPU	Graphics Processing Unit
HDF5	Hierarchical Data Format version 5
LRP	Layer-Wise Relevance Propagation
MLO	Medio-Lateral Oblique (mammographic view)
MRI	Magnetic Resonance Imaging
PNG	Portable Network Graphics
ReLU	Rectified Linear Unit (activation function in neural networks)
ResNet50	Residual Neural Network (50-layer deep learning model)
ROC Curve	Receiver Operating Characteristic Curve
TN	True Negative
TP	True Positive
